# Supplementary material for: COZOID: contact zone identifier for visual analysis of protein-protein interactions
Source: BMC Bioinformatics. 2018 Apr 6;19:125. doi: 10.1186/s12859-018-2113-6 (PMC5889581; doi:10.1186/s12859-018-2113-6)
Supplement: Supplementary file 2 — Software build. Executable binary file of the software tool. (ZIP 118,784 kb) [file 12859_2018_2113_MOESM2_ESM.zip › cozoid/platform/docs/testng-6.5.1-javadoc.zip!/org/testng/ITestNGListenerFactory.html]

ITestNGListenerFactory (TestNG)


---


|  |  |  |
| --- | --- | --- |
| **Overview** \| **Package** \| **Class** \| **Tree** \| **Deprecated** \| **Index** \| **Help** | |  |
| **PREV CLASS**   **NEXT CLASS** | **FRAMES**    **NO FRAMES**     **All Classes** |  |
| SUMMARY: NESTED \| FIELD \| CONSTR \| METHOD | DETAIL: FIELD \| CONSTR \| METHOD |  |


---


## org.testng Interface ITestNGListenerFactory

---

``` public interface ITestNGListenerFactory ```

A factory used to create instances of ITestNGListener. Users can implement this interface
in any of their test classes but there can be only one such instance.

---

| **Method Summary** | |
| --- | --- |
| `ITestNGListener` | `createListener(java.lang.Class<? extends ITestNGListener> listenerClass)`             Create and return an instance of the listener class passed in parameter. |

**Method Detail**

### createListener

```
ITestNGListener createListener(java.lang.Class<? extends ITestNGListener> listenerClass)
```

:   Create and return an instance of the listener class passed in parameter. Return null
    if you want to use the default factory.


---


|  |  |  |
| --- | --- | --- |
| **Overview** \| **Package** \| **Class** \| **Tree** \| **Deprecated** \| **Index** \| **Help** | |  |
| **PREV CLASS**   **NEXT CLASS** | **FRAMES**    **NO FRAMES**     **All Classes** |  |
| SUMMARY: NESTED \| FIELD \| CONSTR \| METHOD | DETAIL: FIELD \| CONSTR \| METHOD |  |


---
